# Dataset of complete genome assembly and analysis of mycobacterium tuberculosis strain SIT745/EAI1-MYS

**DOI:** 10.1016/j.dib.2020.105949

**Published:** 2020-06-30

**Authors:** Mohammad Abdullah, Siti Suraiya, Suharni Mohamad, Azian Harun

**Affiliations:** a Medical Microbiology and Parasitology Department, School of Medical Sciences, Universiti Sains Malaysia, Kelantan, Malaysia; b Infection Control Unit, Hospital Universiti Sains Malaysia, Malaysia; c School of Dental Sciences, Universiti Sains Malaysia, Kelantan, Malaysia

**Keywords:** *Mycobacterium tuberculosis*, Genome assembly and annotations, Phylogenetic analysis, Comparative genomics, Kelantan, Malaysia

## Abstract

In this dataset, we report the genome assembly and data analysis of *Mycobacterium tuberculosis* strain SIT745/EAI1-MYS*.* Previously, this strain was isolated from a Malaysian patient with extra-pulmonary tuberculosis, and identification of this strain is done by spoligotype patterns with fifteen known Shared International Type (SITs). Further analysis showed that this strain has a remarkable phylogeographical specificity for Malaysia. Based on the National Center for Biotechnology Information (NCBI) nucleotide database information, the complete genome consists of 150 contigs with various sequence lengths and was not assembled. In this assembly, the aforementioned contigs along with reference sequence from *Mycobacterium tuberculosis* strain H37Rv and *Mycobacterium bovis* strain AF2122/97 was used for gap closures, were assembled into a single circular chromosome length of approximately 4.42 Mega bases (Mb) with an average GC content of 65.6%. The single circular chromosome was shown to contain 4,009 protein-coding sequences, 3 ribosomal RNAs, 45 transfer RNAs, and 12 superclasses distributed with 277 subsystems which constitute nearly 1900 genes, respectively. The genome information will provide fundamental knowledge of this organism as well as insight for understanding genomic and proteomic profiling, phylogenetic relationship.

Specifications table

**Table:** 

Subject	Immunology and Microbiology
Specific subject area	Microbiology, Genomics
Type of data	Genome assembly, Tables and Figures
How data were acquired	NCBI accession number: LUDZ01000001-LUDZ01000150 Contigs assemble: SnapGene V.5.0.5 platform Genome annotation: NCBI Prokaryotic Genome Annotation Pipeline (PGAP) Subcellular localization prediction: TBpred Prediction server Functional categorization prediction: Tuberculist database Superclasses and their corresponding subsystems features: PATRIC server Sequence alignment with Bio Edit version 7.2.5 Phylogenetic analysis and Maximum Likelihood phylogenetic tree with MEGA version 10.1 and PhyML respectively
Data format	Raw and analyzed.
Parameters for data collection	Data (contigs) was collected from NCBI accession No LUDZ01000001-LUDZ01000150
Description of data collection	Complete genome assembly of *M.tuberculosis* SIT745/EAI1-MYS is done using contigs. BLAST was performed on contigs, corrections and gaps between the sequences are replaced with the reference genome sequence of *M. tuberculosis* strain H37Rv and *M. bovis* strain AF2122/97, and genome annotation
Data source location	Hospital Universiti Sains Malaysia (Hospital USM), Kelantan, Malaysia
Data accessibility	Within this article and at http://dx.doi.org/10.17632/9kgt46cpdh.1
Related research article	Suraiya S, Semail N, Ismail M, Abdullah J. Complete genome sequence of *Mycobacterium tuberculosis* clinical isolate spoligotype SIT745/EAI1-MYS, Genome Announc. 4 (2016)

## Value of the Data

• This data will be useful to the clinicians and researchers working on *Mycobacterium tuberculosis* strain.
• The data will give insight into proteomic profiling analysis, genetic virulence and diversity of the *M. tuberculosis* strain SIT745/EAI1-MYS.
• The data can be useful to understand the relation between *M. tuberculosis* strains from Kelantan and other regions of Malaysia. This, in turn, could help to take the necessary steps for the prevention of tuberculosis.

## 1 Data description

**Fig. 1:**
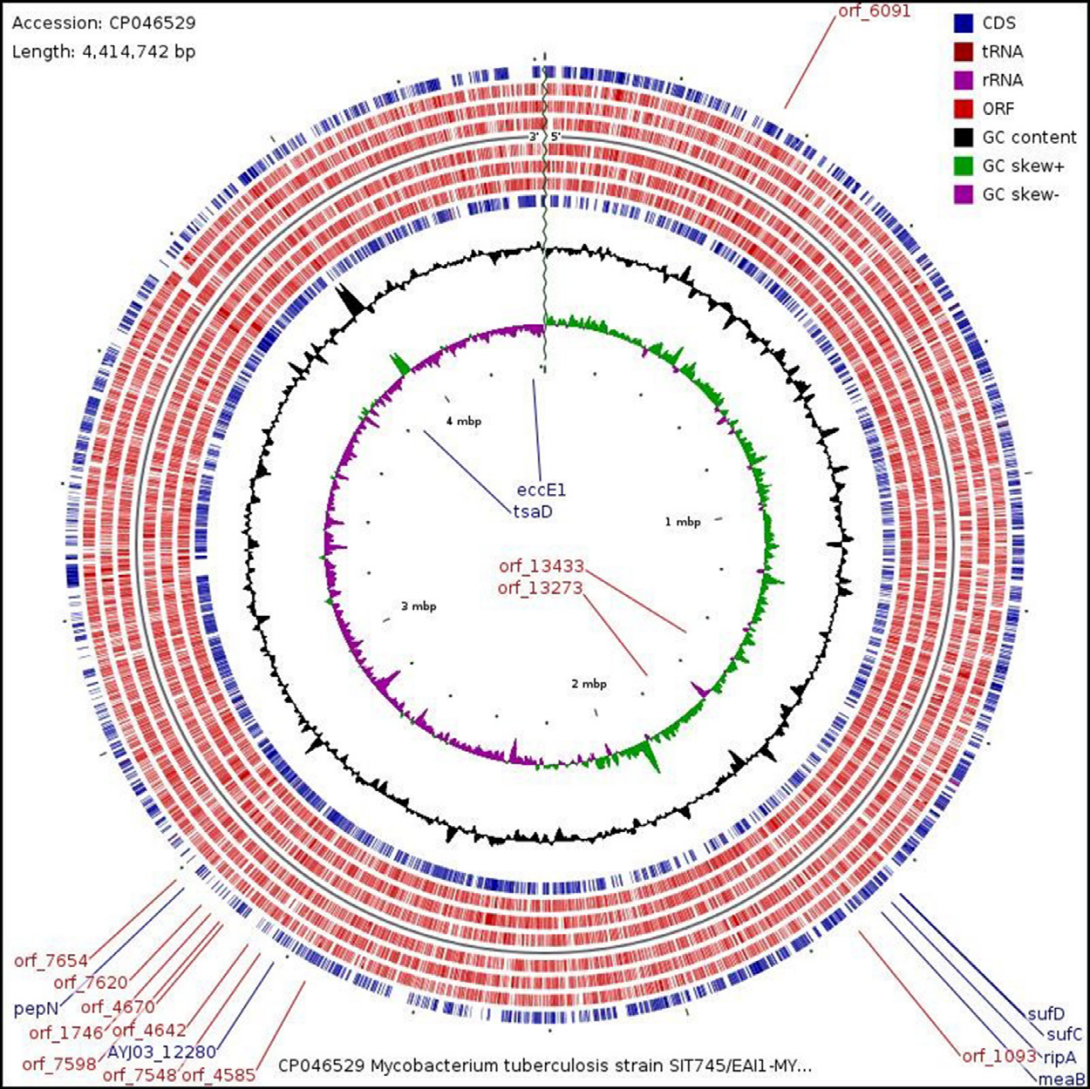
Chromosome features of *Mycobacterium tuberculosis* strain SIT745/EAI1-MYS. Track 1 and 9, coding sequence (CDS - forward and reverse). Track 2–4 and 6–8, open reading frame (forward and reverse). Track 5, Chromosomal sequence direction (5′−3′). Track 9, GC content. Track 10, GC skew + (green) and GC skew- (purple). Track 11, genome size.(For interpretation of the references to color in this figure legend, the reader is referred to the web version of this article.)

**Fig. 2:**
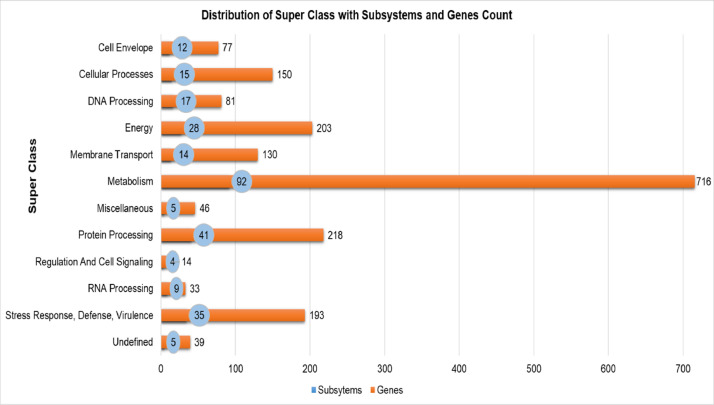
The 12 Superclass distribution to the number of subsystems and genes of *M. tuberculosis* strain SIT745/EAI1-MYS is generated from the PATRIC annotation server.

**Fig. 3:**
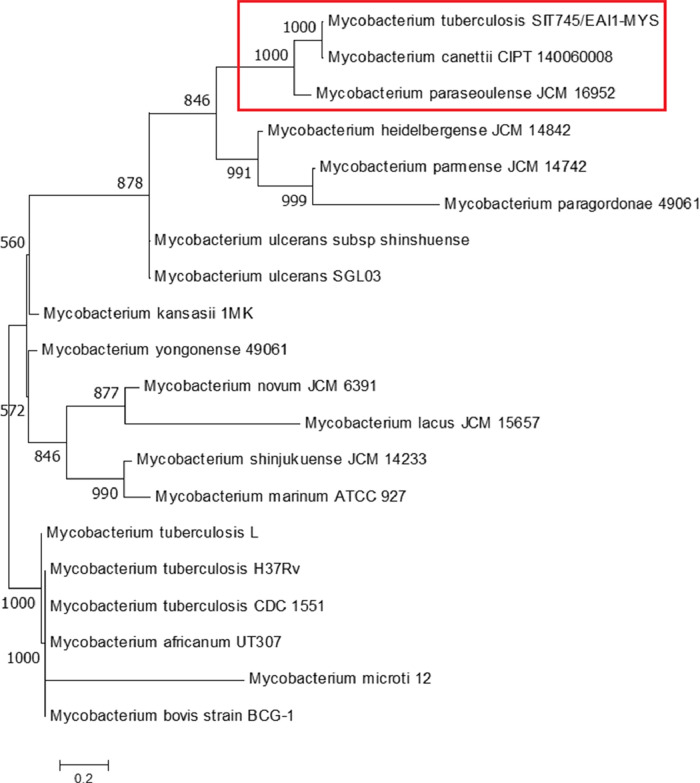
Comparative phylogenetic analysis of strain SIT745/EAI1-MYS. ML phylogenetic tree is based on the concatenated dataset; the analysis is based on an alignment of 10,608 nucleotide positions from 20 isolates. GTR+*I* + *G* evolutionary model was used to generate the ML tree. Bootstrap values >500 for ML are shown at the branches. This strain is clustered with other strains CIPT 140060008 and JCM 16952 (high support −1000) (red rectangular box).(For interpretation of the references to color in this figure legend, the reader is referred to the web version of this article.)

Mycobacterium tuberculosis is an acid-fast bacillus considered to be the causative agent for tuberculosis (TB) [1]. *M. tuberculosis* strain SIT745/EAI1-MYS was found to be the second most predominant strain in the Kelantan region, Malaysia [2]. The strain was identified based on the spoligotype patterns with fifteen known Shared International Type (SITs) [3]. Previously, this strain was isolated from a Malaysian patient with extra-pulmonary tuberculosis who was admitted to Hospital Universiti Sains Malaysia (Hospital USM). Whole-genome shotgun sequencing using Illumina MiSeq platform was performed on the genomic DNA from a 3-week old culture. The sequences were deposited under the accession no. LUDZ00000000 at DDBJ/ENA/GenBank by our research group [4]. The sequence assembly of reads generated 150 contigs (NCBI accession number: LUDZ01000001-LUDZ01000150) which ranges from 490 base pairs (bp) to 183,063 bp with an average of 29,000 bp. The data in this article describes the complete genome assembly and data analysis of Mycobacterium tuberculosis strain SIT745/EAI1-MYS. Fig. 1 describes the features of a single circular chromosome of the aforementioned strain using CGView. Fig. 2 describes the 12 superclass distribution, its subsystems, and genes of *M.tuberculosis* strain SIT7/EAL1-MYS which is generated from the PATRIC annotation server. Fig. 3 describes the phylogenetic analysis with a sequence length of 10,608 base pairs of Mycobacterium tuberculosis SIT745/EAI1-MYS with 19 more isolates. Table 1 describes the complete genome assembly statistics and genome content of the aforementioned strain. Table 2 describes the list of twelve superclasses and its subsystems features**.** Table 3 describes the genomic features of six *Mycobacterium* strains. The dataset used to assemble a complete sequence is of 150 contigs (NCBI accession number: LUDZ01000001-LUDZ01000150). Supplementary material 1 describes the subcellular localization and functional categorization of the protein-coding sequences (CDS) which was predicted using the TBpred Prediction server and Tuberculist database. Supplementary material 2 describes the list of 12 superclasses with a distribution of 277 subsystems and 1900 genes. Supplementary material 3 describes the list of 20 *M.tuberculosis* strains with NCBI accession number which were used to construct phylogenetic analysis. Supplementary material 4 describes the sequence length of 10,608 base pairs used to construct the Maximum Likelihood phylogenetic tree. Supplementary material 5 describes the 12 Superclass list used for creating Fig. 2 chart. Raw data and supplementary materials available at http://dx.doi.org/10.17632/9kgt46cpdh.1.

**Table 1:** Assembly statistics and genome content for M. tuberculosis SIT745/EAI1-MYS.

Assembly Statistics		Genome Content	
Total length of sequences (bp)	4414,742	Overall GC (%)	65.6
Total number of contigs	150	Total genes	4093
150 contigs length (bp)	4371,919	CDS	4009
Average length	29,146	Pseudogenes	84
Smallest contig (bp)	490	Total RNAs	51
Largest contig (bp)	183,063	tRNAs	45
Coverage sequencing	194X	Non-coding RNA (ncRNAs)	3
N50 contig (bp)	68,502	3 rRNA's (5 s,16 s, 23 s)	1 each
L50 contig	20	NCBI Accession No	CP046529

**Table 2:** List of 12 Super Classes and its corresponding subsystems features.

Super Class	Subsystems features
Cell Envelope	Cell Envelope, Capsule and Slime layer
Cellular Processes	Cell Cycle, Cell Division and Death, Prokaryotic cell type differentiation
DNA Processing	DNA Processing
Energy	Energy and Precursor Metabolites Generation, Respiration
Membrane Transport	Membrane Transport
Metabolism	Amino Acids and Derivatives, Carbohydrates, Cofactors, Vitamins, Prosthetic Groups, Fatty Acids, Lipids, and Isoprenoids, Iron acquisition and metabolism, Metabolite damage and its repair or mitigation, Nitrogen Metabolism, Nucleosides and Nucleotides, Phosphate Metabolism, Sulfur Metabolism
Miscellaneous	Miscellaneous
Protein Processing	Protein Fate (folding, modification, targeting, degradation), Protein Synthesis
Regulation and Cell Signaling	Regulation and Cell Signaling
RNA Processing	RNA Processing
Stress Response, Defense, Virulence	Stress Response, Defense, Virulence
Undefined	Clustering-based subsystems, Fatty Acids, Lipids, and Isoprenoids

**Table 3:** Comparison of six *Mycobacterium* isolates genomic features based on Phylogeny analysis.

Species	length (bp)	GC content (%)	Predicted ORFs	rRNA genes	tRNA genes	NCBI Reference Sequence
*M. tuberculosis SIT745/EAI1-MYS*	4414,742	65.6	3984	3	45	NZ_CP046529.1
*M.canettii CIPT 140,010,059*	4482,059	65.6	3952	3	45	NC_015848.1
*M.paraseoulense* strain JCM 16,952	6085,955	67.9	5539	3	46	NZ_AP022619.1
*M.parmense* strain JCM 14,742	5952,912	68.4	5275	3	48	NZ_AP022614.1
*M.heidelbergense* strain JCM 14,842	5050,576	67.9	4484	3	45	NZ_AP022615.1
*M.paragordonae* strain 49,061	6730,319	67.0	6310	3	48	NZ_CP025546.1

## 2 Experimental design, materials, and methods

### 2.1 Data pre-processing, genome assembly

Prior to assembly process, the aforementioned contigs were subjected to BLAST (https://blast.ncbi.nlm.nih.gov/Blast.cgi) [5] to identify the related sequences along with terminal ends for each contig. The contigs were assembled using SnapGene V.5.0.5 platform (https://www.snapgene.com/) [6] and resulted in a sequence length of 4371,919 bp with gaps in sequence (approximately 42,823 bp). Initially, corrections for possible misassembled reads and certain gap closures were achieved through mapping against published reference genome sequence of M. tuberculosis strain H37Rv (1st reference sequence, GenBank Accession No. NC_000962) [7]. The sequence similarities above 80% and an alignment of contig adjacent regions of nearly 200 bp with the reference sequence are taken into consideration for gap closure. Later, we noticed the assembled sequence (i.e. contigs + 1st reference sequence) is incomplete with gaps. Thus, the left gaps are further replaced with the 2nd reference genome sequence (i.e., M.bovis strain AF2122/97, GenBank Accession No: NC_002945). Finally, a combination of 150 contigs (i.e., 4371,919 bp) and gap closures (i.e., 42,823 bp) which were incorporated with aforementioned reference 1st and 2nd genomes was assembled into a single circular chromosome. The resulted chromosome has a sequence length of 4414,742 base pairs (bp) or approximately 4.42 Mega bases pairs (Mb) with 65.6%. GC content. The map of a single circular chromosome is generated using CGView (http://wishart.biology.ualberta.ca/cgview/) [8] (Fig. 1).

### 2.2 Assembly statistics and genome content

The genome annotation for the *M. tuberculosis* strain SIT745/EAI1-MYS was done using the NCBI Prokaryotic Genome Annotation Pipeline (PGAP), (https://www.ncbi.nlm.nih.gov/genome/annotation_prok/) [9]. The complete sequence showed a total of 4093 genes out of this 4009 are protein-coding sequences (CDS), 3 ribosomal RNAs (rRNA), and 45 transfer RNAs (tRNA). The assembly statistic and genome content are shown in Table 1.

## 3 Genome annotations

The subcellular localization of the CDS was predicted using the TBpred Prediction server (http://crdd.osdd.net/raghava/tbpred/) [10], and Functional categorization was predicted and classified based on Tuberculist database (https://mycobrowser.epfl.ch/) [11] (supplementary material 1). Superclasses and their corresponding subsystems features were analyzed using the PATRIC server (https://www.patricbrc.org/) [12]. It revealed that the *M. tuberculosis* strain SIT745/EAI1-MYS genome showed a total of 12 superclasses such as Cell Envelope, Cellular Processes, DNA Processing, Energy, Membrane Transport, Metabolism, Miscellaneous, Protein Processing, Regulation, Cell Signaling, RNA Processing, Stress Response, Defense, Virulence, Undefined. A total of 277 subsystems with 1900 genes were distributed among 12 superclasses (supplementary material 2). The subsystem's features for each superclass is shown in Table 2. Similarly, the distribution of subsystems and genes for each superclass is shown in Fig. 2.

## 4 Phylogeny analysis with concatenated dataset

The phylogenetic relationship was addressed between 20 *M.tuberculosis* strains (supplementary material 3) from a multi-gene sequence with an alignment sequence length 16S rRNA (1420 bp), *recA* (1056 bp), *rpoB* (7509 bp), *sodA* (623 bp) respectively, were used to concatenated to a sequence length of 10,608 bp (supplementary material 4). BioEdit version 7.2.5 [13] was used to align the nucleotide sequences using CLUSTAL W. The evolutionary model was chosen from the MEGA (Molecular Evolutionary Genetics Analysis) version 10.1 (i.e., models option-find best DNA/protein models) and the chosen model is GTR+*I* + *G* [14]. Maximum Likelihood (ML) phylogenetic tree was constructed using PhyML with the aforementioned chosen model [15]. Bootstrap analysis of 1000 replicates was used to test the robustness of ML tree topologies (Fig. 3).

## 5 Comparison of six clustered mycobacterium isolates

Based on the Phylogenetic analysis, the comparison of genomic features between the closely clustered six *Mycobacterium* isolates is shown in Table 3.

## Declaration of Competing Interest

The authors declare that they have no known competing financial interests or personal relationships that could have appeared to influence the work reported in this paper.

## Appendix Supplementary materials

Image, application 1

Image, application 2

Image, application 3

Image, application 4

Image, application 5

Image, application 6

Image, application 7
