# Supplementary material for: Dataset of complete genome assembly and analysis of mycobacterium tuberculosis strain SIT745/EAI1-MYS
Source: Data Brief. 2020 Jun 30;31:105949. doi: 10.1016/j.dib.2020.105949 (PMC7339031; doi:10.1016/j.dib.2020.105949)
Supplement: Supplementary file 4 [file mmc4.pdf]

Supplementary Material 3

No	Species	Strain	GenBank Accession No
1	Mycobacterium tuberculosis	CDC 1551	AE000516
2	Mycobacterium ulcerans	subsp.shinshuense	AP017624
3	Mycobacterium marinum	ATCC 927	AP018496
4	Mycobacterium novum	JCM 6391	AP022562
5	Mycobacterium shinjukuense	JCM 14233	AP022575
6	Mycobacterium lacus	JCM 15657	AP022581
7	Mycobacterium parmense	JCM 14742	AP022614
8	Mycobacterium heidelbergense	JCM 14842	AP022615
9	Mycobacterium paraseoulense	JCM 16952	AP022619
10	Mycobacterium tuberculosis	H37Rv	CP003248
11	Mycobacterium microti	12	CP010333
12	Mycobacterium bovis	BCG-1	CP011455
13	Mycobacterium africanum	UT307	CP014617
14	Mycobacterium yongonense	Asan 36527	CP015965
15	Mycobacterium kansasii	1MK	CP019883
16	Mycobacterium paragordonae	49061	CP025546
17	Mycobacterium tuberculosis	L	CP044345
18	Mycobacterium tuberculosis	SIT745/EAI1-MYS	CP046529
19	Mycobacterium canettii	CIPT 140060008	FO203507
20	Mycobacterium ulcerans	SGL03	LR135168
